# Pyrolysis of Dutch mixed plastic waste: Lifecycle GHG emissions and carbon recovery efficiency assessment

**DOI:** 10.1177/0734242X241306605

**Published:** 2024-12-31

**Authors:** Juraj Petrík, Homer C. Genuino, Gert Jan Kramer, Li Shen

**Affiliations:** 1 Copernicus Institute of Sustainable Development, Utrecht University, Utrecht, Netherlands; 2 Sustainable Process Technology, Faculty of Science and Technology, University of Twente, Enschede, Netherlands; 3 National Test Centre Circular Plastics (NTCP), Heerenveen, Netherlands

**Keywords:** LCA, DKR-350, circularity, chemical recycling, plastics recycling, environmental impacts

## Abstract

Plastic production and consumption contribute to climate change and the depletion of non-renewable fossil resources, necessitating a shift towards a circular economy. This study explored the potential of pyrolysis as a novel approach to managing plastic waste and achieving plastic circularity in the Netherlands. Specifically, we focused on the pyrolysis of DKR-350, a low-quality mixed-plastic sorting residue. Using the life cycle assessment framework, we analysed DKR-350 pyrolysis, based on empirical data from pilot-scale trials, from two perspectives depending on the system’s primary function: waste management or naphtha production. We also considered the impacts of pyrolysis feedstock pre-treatment, including washing. Our findings demonstrated that pyrolysis of DKR-350, with lifecycle greenhouse gas (GHG) emissions of 876 kg CO_2_ eq. per 1000 kg pyrolysed unwashed DKR-350, can offer significant environmental benefits compared to incineration, resulting in a 28%–31% reduction in lifecycle GHG emissions. Sensitivity analysis showed the potential for achieving a 39%–65% reduction in GHG emissions by 2030, with lifecycle GHG emissions representing a mere 470 kg CO_2_ eq. per 1000 kg pyrolysed unwashed DKR-350 for the best sensitivity case. Lastly, we analysed the carbon recovery efficiency – a potential circularity indicator based on substance flow – resulting in 38%–55% of recovered carbon in pyrolysis oil, the system’s main product from a lifecycle perspective.

## Introduction

Plastics play a vital role in the global economy due to their chemical stability, enabling their application in diverse products, from food packaging to high-performance materials (Jehanno et al., 2022). In 2021, global plastic production amounted to nearly 391 million metric tonnes, with approximately 90% derived from virgin fossil-based sources (Plastics Europe, 2022). However, plastic is associated with adverse environmental impacts, including plastic waste pollution, depletion of fossil resources and greenhouse gas (GHG) emissions throughout manufacturing. Global GHG emissions from the plastic sector are a dominant contributor to climate change, accounting for approximately 1.7 gigatonnes (Gt) of CO_2_ eq. in 2015. With the increasing demand for plastics, this contribution is projected to rise to 6.5 Gt CO_2_ eq. by 2050 (Zheng and Suh, 2019). Plastic production is responsible for 20% of GHG emissions from the chemical sector in the European Union (EU), and the incineration of plastic waste alone results in 50–80 metric tonnes CO_2_ emissions annually (European Environment Agency, 2021).

The transition from a linear to a circular economy presents a potential solution to mitigate GHG emissions from the plastics sector and its waste management (Ellen MacArthur Foundation, 2019; Meys et al., 2021). The circular economy principles are based on the reduction of waste and pollution by minimising material use (reduce), preserving existing materials in use (reuse), enhancing their recyclability (recycle) and utilising regenerative natural resources (e.g. biomass) (Ellen MacArthur Foundation, 2013). On the contrary, only 9% of plastic waste is recycled globally, and if the business-as-usual trend continues, a mere 12% of plastics are projected to be recycled by 2060 (OECD, 2022). International communities and national governments are implementing various circular strategies to reverse this trend. For instance, the European Commission set a target to recycle at least 55% of plastic packaging waste by 2030 (European Commission, 2018). The Netherlands has set an ambitious goal to reduce the use of fossil raw materials for plastics by 50% by 2030 and substitute them with circular raw materials (MIWM, 2023). Nonetheless, achieving circularity and reaching net-zero GHG emissions by 2050 in the EU or the Netherlands necessitates significant transformation within the plastic sector and waste management practices.

In the Netherlands, plastic packaging waste is sorted into five streams according to the quality standards of German Plastic Recycling (Deutsche Kunststoff Recycling, DKR). The four DKR streams consist of mono polymers such as polyethene terephthalate (PET, DKR-328), polyethene (PE, DKR-329), polypropylene (PP, DKR-324) and foils (DKR-310). In contrast, DKR-350, the fifth and largest stream, comprises a mixture of plastics after sorting out easily recyclable mono-plastic fractions. However, DKR-350 is not recycled or has low recycling value due to high contamination and property variability (Genuino et al., 2022). Despite considerable sorting and recycling efforts in the Netherlands, incineration with energy recovery remains the primary end-of-life waste management option regarding plastic packaging. According to Lobelle et al. (2024), in the Netherlands, 229 kt of household packaging waste was incinerated with energy recovery in 2017, representing 57% of the total household packaging waste brought to market.

Recent developments and research in the circularity field and shortcomings of current waste management options showed a need for a new approach to managing plastic waste. Among the novel recycling methods, pyrolysis is a promising waste management solution for DKR-350 and other contaminated mixed plastic waste streams currently incinerated. In pyrolysis, plastics are thermochemically converted to a valuable liquid product known as pyrolysis oil. Consequently, pyrolysis oil could be upgraded and substitute naphtha, a primary fossil fuel feedstock for plastics production. Potentially, pyrolysis of plastic waste could reduce the reliance on fossil resources, mitigate GHG emissions and improve the overall circularity of the sector (Kusenberg et al., 2022).

Numerous studies have investigated lifecycle GHG emissions associated with different end-of-life options for plastics, including pyrolysis (Arena et al., 2023; Benavides et al., 2017; Faraca et al., 2019; Keller et al., 2022; Schwarz et al., 2021; Volk et al., 2021; Yadav et al., 2023; furthermore, see reviews by Antelava et al. (2019) and Pires Costa et al. (2022)). As reported, landfilling plastic waste often emerges as the preferred option for end-of-life scenarios based on lifecycle GHG emissions (Stegmann et al., 2022). However, landfilling combustible waste is prohibited in the Netherlands (Rijkswaterstaat, 2019) and will be phased out in the EU. Furthermore, it does not eliminate the dependence on primary fossil feedstock. Studies also indicate that mechanical recycling of plastics yields significant reductions in lifecycle GHG emissions compared to incineration (Arena and Ardolino, 2022; Civancik-Uslu et al., 2021; Jeswani et al., 2021; Schwarz et al., 2021; Shen et al., 2010). However, the application of mechanical recycling is predominantly limited to single-polymer plastics, and the recycling of contaminated mixed plastics (such as DKR-350) frequently results in downgraded material quality in terms of mechanical properties (e.g. strength and stretch), optical properties (e.g. transparency and colourlessness) or chemical purity (e.g. free of contaminants) (Lange, 2021).

A growing body of literature reports lifecycle GHG emissions from plastic waste pyrolysis. Several studies reported that these emissions are significantly lower than the incineration of mixed plastic waste with energy recovery (Arena et al., 2023; CE Delft, 2019; Civancik-Uslu et al., 2021; Gear et al., 2018; Jeswani et al., 2021; Meys et al., 2020; Qureshi et al., 2020). However, the results of these studies are often sensitive to parameters such as feedstock composition, system boundaries, geographical location and allocation and system credits within the assessment. For example, Jeswani et al. (2021) estimated lifecycle GHG emissions per metric tonne of pyrolysed plastic waste in Germany to be approximately 0.7 tonne CO_2_ eq., whereas CE Delft (2019) reported net-negative impacts for pyrolysis of 1 metric tonne of mixed plastic waste in the Netherlands.

Besides producing naphtha-like pyrolysis oils from mixed plastic wastes, many new developments in the field focus on other waste types or valorising pyrolysis products. For example, Tsangas et al. (2024) studied the environmental impacts of producing pyrolysis oil from waste tyres and its utilisation for electricity generation. In other examples, low-quality pyrolysis oils were upgraded via catalytic steam reforming (Huang et al., 2021) or other pyrolysis by-products valorised via carbon capture from non-condensable pyrolysis gases and converted to multi-walled carbon nanotubes, further utilised as an electrode material or lubricant additives (Ahamed et al., 2020; Veksha et al., 2022).

Despite the considerable research on pyrolysis life cycle assessment (LCA), several notable gaps remain in the existing literature. Firstly, many of the reviewed papers have relied on theoretical models and pyrolysis simulations using data from literature sources or laboratory-scale experiments, focusing primarily on the pyrolysing waste feedstock of a single polymer. Only a few papers investigated the pyrolysis of actual post-consumer contaminated mixed plastic waste at pilot or industrial scales. Secondly, many studies lack comprehensive information regarding process conditions (such as temperature, pressure and residence time), reactor type, catalytic or non-catalytic nature of the process and feedstock composition. Thirdly, to our knowledge, no previous research has investigated the potential benefits of pyrolysis feedstock pre-treatment, particularly the washing process of mixed plastic waste, on overall lifecycle GHG emissions. Washing pretreatment can improve the quality of pyrolysis oil by effectively reducing surface contaminants, residual substances and non-plastic materials in the feedstock. Thus, it may be an essential step to produce high-quality pyrolysis oils (Genuino et al., 2023). However, this benefit comes with the trade-off of increased energy demand for the washing process, and the impact of this trade-off is not well-documented in the literature. For instance, Veksha et al. (2022) studied the effect of washing marine plastic litter on the calorific value of the feedstock and composition of pyrolysis products but did not compare lifecycle impacts for unwashed and washed samples. Lastly, most existing studies have focused on conducting LCAs of pyrolysis primarily from a perspective of waste management technology or, in some cases, a perspective of material recovery. Only Jeswani et al. (2021) encompassed a goal-oriented LCA that considered multiple perspectives, including waste management, product recovery and a combination of the two perspectives.

This study aims to address the gaps mentioned above. Firstly, in the Dutch context, we investigate lifecycle GHG emissions associated with the pyrolysis of sorted post-consumer mixed plastic packaging waste, known as DKR-350. This analysis is based on empirical data measured from pilot-scale pyrolysis trials using a real, contaminated and difficult-to-recycle plastic waste fraction. Secondly, our study aims to provide comprehensive information regarding the pyrolysis process. Additionally, we provide a detailed inventory analysis, including the composition of pyrolysed waste fractions. Thirdly, our study’s significant focus is understanding the impacts of DKR-350 pre-treatment through washing. Fourthly, we present a goal-oriented LCA that considers two distinct perspectives: waste management and naphtha production. These perspectives are compared to their reference benchmarks: incineration with energy recovery and fossil-based naphtha production. Lastly, in addition to evaluating the lifecycle GHG emissions, we apply the overall carbon recovery efficiency – a potential indicator based on substance flow – to address climate change and material circularity challenges. With the indicator, we analyse the overall carbon recovery efficiency of the pyrolysis system, including the carbon losses as trade-offs, and explore implications for plastics circularity.

## Material and methods

### Goal and scope

This study aims to evaluate lifecycle GHG emissions throughout the lifecycle of DKR-350, recycled via innovative non-catalytic pyrolysis technology. The results aim to provide insights into a potential reduction of lifecycle GHG emissions and to identify opportunities for further technological advancements. The assessment was conducted in two scenarios based on whether the DKR-350 stream was subjected to washing (the washed case) or not (the unwashed case) during the pyrolysis feedstock pre-treatment steps. The findings were compared to the conventional waste management approach for DKR-350, which involved incineration with energy recovery.

The LCA carried out in this study followed the ISO 14040 and 14044 standards (ISO, 2006a, 2006b). In the LCA, we adopted a cradle-to-gate approach, starting with a sorted DKR-350 stream at the beginning of the lifecycle and ending with the final products, leaving the system at a production facility. The cut-off method was employed for the DKR-350 waste stream to exclude the environmental impacts associated with its previous lifecycle. The geographical scope of the study was limited to the Netherlands, and the temporal scope represented the production as of 2020–2022. The technical scope encompassed a combination of technologies with varying technology readiness levels, predominantly at average market technology levels, and with the DKR-350 pyrolysis unit operated at a pilot-scale level. Infrastructure and transportation impacts were excluded from the assessment.

**Figure 1.**
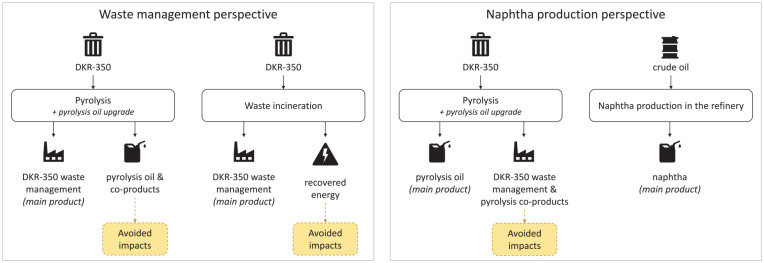
Framework for the LCA perspectives defined for the study: waste management perspective (left) and naphtha production perspective (right). LCA: life cycle assessment.

Furthermore, this assessment recognised that pyrolysis serves two primary functions, namely, (1) waste management and (2) pyrolysis oil production, where pyrolysis oil has properties similar to fossil naphtha, used as a feedstock for steam-cracking and the production of plastics (see Figure 1). Thus, the assessment was carried out from two perspectives, depending on whether waste management or naphtha production was considered the primary function of the DKR-350 pyrolysis. The results of these two perspectives were treated as separate LCA studies.

#### Waste management perspective

**Figure 2.**
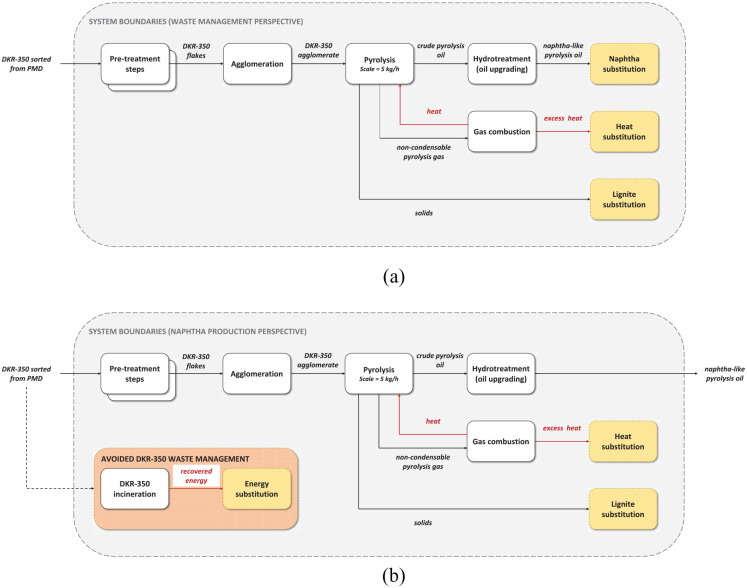
System boundaries defined for the study: (a) waste management perspective; (b) naphtha production perspective.

In the waste management perspective, pyrolysis was considered as technology replacing conventional waste management options for DKR-350. Thus, the functional unit was defined as waste management of 1000 kg DKR-350. The results were compared with a reference system of DKR-350 incineration with energy recovery. The system boundaries (see Figure 2(a)) of the pyrolysis system included upgrading the main pyrolysis product, pyrolysis oil, to naphtha-like quality oil by removing contaminants via hydrotreatment. Pyrolysis products and recovered energy were credited within the system boundaries.

#### Naphtha production perspective

In the naphtha production perspective, pyrolysis was considered as technology primarily producing pyrolysis oil and replacing the production of fossil naphtha in oil refineries. Thus, pyrolysis oil was upgraded (similarly as in the waste management perspective) and compared to a reference system of naphtha production from crude oil. The functional unit is defined as producing of 1000 kg naphtha-quality liquid hydrocarbon mixture. Additionally, the pyrolysis system was credited for pyrolysis co-products and avoided GHG emissions from the DKR-350 waste management (see the system boundaries in Figure 2(b)).

### Life cycle inventory

Data for the LCA inventory were primarily collected through pilot-scale pyrolysis trials of DKR-350, carried out within the CPI project (CP-50-02). Thus, data were gathered from primary sources, including site visits and interviews with operators. Additional data were gathered from secondary sources such as (industry) reports and scientific literature. The following sections outline the key data and assumptions for the lifecycle processes: DKR-350 feedstock, pre-treatment, agglomeration, pyrolysis, gas combustion, hydrotreatment, incineration and naphtha production. A detailed life cycle inventory can be found in the Supplemental Material.

#### DKR-350 feedstock

DKR-350 is a mixed plastic packaging waste stream sorted from the PMD stream (plastic packaging, metal packaging and drink cartons). The DKR-350 feedstock used in the pyrolysis trials has been sorted from the Dutch household PMD waste stream collected in December 2021 and January 2022. The dominant fraction of DKR-350 is polyolefins, consisting of 42%–45% PE and 17%–18% PP. Other significant fractions are 7%–11% PET and 8% paper and cardboard. Furthermore, DKR-350 is contaminated by impurities, such as food residues (i.e. organic fraction) and metals. The moisture content of DKR-350 was determined to be 14 wt.% (NTCP, 2020). Additional information on the composition of the materials, higher heating value (HHV) and carbon content can be found in the Supplemental Material. It is assumed that the DKR-350 waste stream entered the system without any environmental burden.

#### Pre-treatment

##### Unwashed case

In the unwashed case, the only pre-treatment step assumed was shredding of the DKR-350 waste stream into flakes. The waste is shredded using a Shini granulator SG-2336 (NTCP, 2020) with an estimated electricity consumption of 0.077 kWhour per 1 kg of processed waste. No (target) material losses were assumed during the shredding process.

##### Washed case

In the washed case, DKR-350 was first shredded, and then the flakes were washed. The washing process followed the ‘cold wash + hot wash’ protocol previously used in lab-scale pyrolysis trials (Genuino et al., 2022). The specific energy requirement for washing was estimated at 0.1 kWhour of electricity per kg of waste (based on a plastic washing line with a capacity of 1000 kg hour^−1^). Additional utilities related to the washing process, such as detergent and wastewater treatment, are included in the LCA.

#### Agglomeration

The agglomeration step homogenised DKR-350 flakes and increased their bulk density, making them a suitable feedstock for pilot-scale pyrolysis reactor operation. The agglomeration line operates at 170°C and has an electricity consumption of approximately 0.55–0.75 kWhour per 1 kg of DKR-350 flakes. A median value of 0.65 kWhour per kg of DKR-350 flakes was used in the baseline modelling. No material losses were assumed from the agglomeration process except for moisture (removed due to high operational temperature) and metals (removed by magnet).

#### Pyrolysis

The pilot-scale pyrolysis of DKR-350 was described in detail by Genuino et al. (2023). The pyrolysis ran in a continuous-mode, fluidised bed reactor with a feed rate capacity of 5 kg hour^−1^ and residence time of around 2 minutes. The total pyrolysed feedstock used per case (unwashed and washed DKR-350) was 25 kg. The reaction occurred at about 500°C under atmospheric pressure in an inert nitrogen atmosphere. As the specific energy consumption (SEC) was not measured during the pilot-scale trials, we assume a value of 15% of the feedstock calorific input based on empirical data from a pilot-scale pyrolysis reactor reported by Zilva (2022). Alternative studies report lower SEC values, ranging from 3% to 8% (Elordi et al., 2018; Fivga and Dimitriou, 2018; Jeswani et al., 2021). However, these studies often lack information on the reactor’s operating temperature or are based on theoretical process simulation models. Therefore, we opted for a more conservative SEC of the pyrolysis reactor.

In the pilot-scale set-up, a series of condensers and a scrubber were employed to collect maximal pyrolysis oil yield. During the trials, the condensation heat was not utilised. However, in large-scale operations, it is common practice to use the heat from vapours exiting the reaction zone within pyrolysis plants (Papari and Hawboldt, 2018). Theoretically, if we assume that DKR-350 pyrolysis oil vapours have properties similar to those of light oil vapours (Genuino et al., 2022), such as diesel, with a median specific heat capacity of 2.3 kJ (kg·°C) ^−1^, a condensation temperature range of 180°C–350°C, and latent heat of 230 kJ kg^−1^, approximately 960 kJ heat per kg of pyrolysis oil could be recovered when the oil vapours cool down from 500°C to 180°C and condense. By integrating this process with an Organic Rankine Cycle (ORC) with a 15% efficiency for electricity production, approximately 0.040 kWhour of electricity per kilogram of oil could be generated. Furthermore, cooling the condensed pyrolysis oil from 180°C to 20°C would require additional energy for the condenser’s cooling duty. Assuming the condenser’s coefficient of performance is between 3 and 6, the required electricity would be approximately 0.017–0.034 kWhour per kilogram of oil. This demand could be adequately met by the electricity produced from the recovered heat in the ORC. Therefore, we did not account for additional emissions in our LCA model due to condenser energy use.

Pyrolysis product yields, determined in pilot-scale trials, are critical parameters for LCA modelling. Product yield is the quantity of collected pyrolysis product relative to the input feedstock to the pyrolysis reactor, excluding the total system input. Three product fractions were collected: pyrolysis oil, non-condensable gases and solids. Combined yields ranged from 72 wt.% to 80 wt.% in the unwashed and washed cases. In the unwashed case, yields were 40 wt.% for pyrolysis oil, 25.4 wt.% for gas and 6.6 wt.% for solids; in the washed case, these increased to 43.9 wt.%, 28.3 wt.% and 7.5 wt.%, respectively. A portion of the generated products was not collected, hereinafter referred to as the uncollected mass. Achieving a balanced mass closure, summing up to a combined yield of 100%, is frequently challenging in pilot-scale settings compared to scaled-up industrial operations. This challenge arises due to limitations inherent to equipment design, including issues such as clogging of oil or oil/wax within tubes. After pyrolysis, the reactor was subjected to elevated temperatures and flushed with oxygen to identify any residual solid mass. Notably, no discernible carbon emissions were detected in the exhaust gas, providing conclusive evidence that no solid products remained within the reactor. Based on this empirical data, the uncollected mass could theoretically be collected as pyrolysis oil or gas. For modelling in the baseline scenarios, it was assumed that the uncollected mass primarily consisted of pyrolysis oil, yielding 68 wt.% and 64 wt.% in the unwashed and washed cases, respectively (see Table 1 for elemental composition of pyrolysis oils). Furthermore, this uncertain assumption is examined in detail in the sensitivity analysis.

**Table 1.** Elemental composition (CHNSO, wt.%) in mixed pyrolysis oils collected from condenser and scrubber.

Element	Unwashed	Washed
Carbon	87.09	85.82
Hydrogen	11.34	11.95
Nitrogen	0.28	0.18
Sulphur	0.012	0.005
Oxygen	1.27	2.04

#### Gas combustion

In the LCA model, the gas collected from pilot-scale trials was assumed to be combusted in a boiler with a 90% combustion efficiency to recover energy. The HHV of the gas ranged from 38 to 39 MJ kg^−1^, depending on the case (see Table 2). Although energy recovery and heat utilisation did not occur in the pilot-scale pyrolysis set-up, the LCA model assumed that the recovered energy would cover the energy demand of the pyrolysis reactor. According to our model, the heat produced via pyrolysis gas combustion was sufficient to meet the pyrolysis energy consumption requirements. Approximately 6 MJ of heat is needed to pyrolyse 1 kg of DKR-350, based on 15% of the feedstock’s calorific input. The net heat produced from gas combustion ranged between 9 and 10 MJ per 1 kg of pyrolysed waste, which was adequate to cover the pyrolysis reaction’s energy demand. The excess heat recovered from gas combustion was substituted, and credits were given to the pyrolysis system.

**Table 2.** Composition (in wt.%) and calculated HHV (in MJ kg^−1^ gas) of collected pyrolysis gases.

Component	Unwashed	Washed
H_2_	2.23	3.13
CO	6.64	8.06
CO_2_	24.52	22.49
CH_4_	9.99	10.22
Ethylene	15.62	16.99
Ethane	3.58	3.52
Propylene	30.86	28.56
Propane	4.16	3.74
Benzene	2.11	3.04
Toluene	0.29	0.25
**HHV gas (MJ kg** ^−1^ **)**	**37.52**	**38.77**

HHV: higher heating value.

#### Hydrotreatment

Crude pyrolysis oil from the pilot-scale trials contained high elemental contamination from DKR-350 feedstock, specifically, nitrogen, oxygen, chlorine and other (semi-)metals. Additionally, it contained unsaturated and aromatic hydrocarbons formed during the pyrolysis process. To compare pyrolysis oil with fossil naphtha, the oil must be upgraded to naphtha-like quality by removing heteroatoms and metals and saturating unsaturated C–H bonds. This upgrade can be done via catalytic hydrogenation (further referred to as hydrotreatment). Since hydrotreatment of pyrolysis oil was not carried out in this research, we modelled this step theoretically. The amount of hydrogen needed for elemental contaminant removal was estimated based on stoichiometric ratios, assuming the removal of nitrogen, sulphur and oxygen. Additional hydrogen was used to saturate and de-aromatise hydrocarbons in the oil. Furthermore, we assumed electrical energy for pumping and compression based on the process simulation model of hydrogenation of waste cooking oil reported by Barbera et al. (2020). Other impacts of this process were not included. However, we might have overestimated the environmental impact of this process since we did not include heat recovery from the exothermic hydrogenation process. If such recovery would be optimised within the system, the overall impact could be reduced.

#### Incineration

In the waste management perspective, pyrolysis cases (unwashed and washed) were compared to the incineration of DKR-350 with energy recovery as a conventional waste management option. Lifecycle GHG emissions from incineration were estimated based on the carbon content in DKR-350 and the stoichiometric ratio of carbon conversion to carbon dioxide. Average net electrical and net thermal waste-to-energy incineration efficiencies of 15% and 32%, respectively, were based on the average efficiencies of approximately 400 European waste-to-energy plants that are part of the Confederation of European Waste-to-Energy Plants (CEWEP) in April 2021 (CEWEP, 2022). Impacts related to the operation of the waste incinerator were also considered.

#### Fossil-naphtha production

In the naphtha production perspective, the pyrolysis cases (unwashed and washed) lifecycle GHG emissions were compared to conventional naphtha production from crude oil in a refinery. The ecoinvent dataset was used to obtain the data for naphtha production from crude oil.

#### Background data

The data from pyrolysis trials were supplemented with background data from the ecoinvent database version 3.7.1, using cut-off unit process datasets (ecoinvent, 2020). The electricity dataset was updated based on the latest available data for the electricity supply mix in the Netherlands (data were taken from the Netherlands Climate and Energy Outlook 2021 report (PBL, 2021)). GHG emissions from hydrogen production via steam methane reforming in the Netherlands were obtained from Fernández-Dacosta et al. (2019). Assumptions in the LCA inventory were primarily based on literature sources or expert consultations. A detailed overview of the background processes and complete inventory can be found in the Supplemental Material.

#### Multifunctionality and allocation

In this study, the multifunctionality of the system was addressed by system expansion via substitution applied to credit co-products, including upgraded pyrolysis oil, solid fraction from pyrolysis, recovered electricity and heat from waste incineration, excess heat from pyrolysis gas combustion and avoided waste management.

- In the waste management perspective, a system expansion via substitution was applied to the upgraded pyrolysis oil, considering it a co-product alongside waste management. The upgraded oil was assumed to have the same properties as fossil naphtha, resulting in a 1:1 substitution based on mass.
- The solid fraction from pyrolysis was also considered a co-product, and a system expansion via substitution was applied by substituting lignite. Credits for substituted lignite were given to the corresponding system. The energy content was used for the 1:1 substitution, estimating the solids’ HHV based on carbon content and carbon’s HHV, whereas the remaining composition was assumed to be inert.
- The produced electricity was assumed to substitute the production of the average market electricity mix, and credits were calculated accordingly. Similarly, credits were given for avoiding average industrial heat from natural gas combustion. Credits were also provided for excess heat produced via pyrolysis gas combustion, considering the heat utilised in the pyrolysis or hydrotreatment process. The same dataset was used to calculate credits.
- In the naphtha production perspective, the pyrolysis systems were credited with avoided impacts from DKR-350 waste management. System expansion via substitution was applied, and credits were given for avoiding emissions from DKR-350 incineration. However, a loss was accounted for in terms of electricity and heat that would otherwise be recovered from the waste incinerator.

#### Sensitivity analysis

Following the ISO 14040 and 14044 standards for LCA, sensitivity analysis was carried out for sensitive parameters and critical assumptions. For a detailed explanation, see ‘Sensitivity analysis’ under the ‘Discussion’ section.

### Impact assessment and indicators

Our environmental impact assessment focused on analysing lifecycle GHG emissions using the IPCC 2013 GWP 100a V1.03 method (IPCC, 2013).

Although measuring GHG emissions is a valuable indicator to address the environmental sustainability dimension, it falls short in capturing progress towards circularity in waste management systems, such as pyrolysis. The circularity assessment is often conducted using mass-balance-based indicators derived from the material flow analysis framework. These indicators emphasise increasing the input of renewable and recycled resources while reducing waste quantities, which is essential for measuring circularity. However, these indicators have limitations in assessing circularity performance, as they do not account for the extent to which recyclates are reintegrated into the cycle or the material losses incurred during sorting, collection and recycling processes (Corona et al., 2019). To overcome these limitations, measuring the circulation of carbon can serve as a suitable indicator, addressing challenges associated with measuring raw material circulation in open-loop recycling and loops involving chemical recycling technologies that involve material conversion (e.g. from plastic waste to pyrolysis oil and new polymers).

In our study, we assessed the carbon flow of the pyrolysis system, and based on that, we derived a carbon recovery efficiency indicator (*η_C_*) (equation (1)):

(1) $$\eta_{C}\left[\%\right]=\frac{m_{C,\mathrm{oil}}[\mathrm{kg}]}{m_{C,\;\mathrm{feedstock}}\left[\mathrm{kg}\right]+m_{C,\;\mathrm{ind}.\mathrm{proc}.}\left[\mathrm{kg}\right]}*100$$

The carbon recovery efficiency represents a share of carbon recovered in pyrolysis oil (*m_C_*,_oil_) relative to the sum of total carbon input in pyrolysis feedstock (direct carbon flow, *m_C_*,_feedstock_) and the carbon input in indirect processes associated with the studied system (indirect carbon flow, *m_C_*,_ind.proc._). The carbon input in indirect processes is calculated based on the amount of airborne fossil CO_2_ emissions caused by these processes. Carbon emissions other than airborne fossil CO_2_ (e.g. methane) are excluded form the calculation, as airborne fossil CO_2_ emissions constitute more than 99% by weight of all carbon emissions in the studied processes.

Our definition of the carbon recovery efficiency indicator in circularity assessment excludes carbon recovered in pyrolysis gas and solid fractions. These fractions are difficult to reintegrate into material production due to their physical and chemical properties, which limits their suitability for applications beyond energy recovery. The critical distinction compared to pyrolysis oil lies in the potential for material recovery: pyrolysis oil is more likely to be processed into valuable chemical feedstocks, thereby contributing more significantly to the circular economy. In contrast, the solid and gas fractions are inherently more challenging to convert back into material forms, making them more suitable for energy recovery.

Although measuring carbon recovery efficiency may enhance our understanding of circularity degrees and recycling strategy performance, it does not encompass the assessment of environmental trade-offs associated with those strategies. Thus, a comprehensive evaluation of sustainability and circularity necessitates combining the carbon recovery efficiency indicator with LCA indicators, such as lifecycle GHG emissions.

## Results

### Lifecycle GHG emissions

#### Waste management perspective

**Figure 3.**
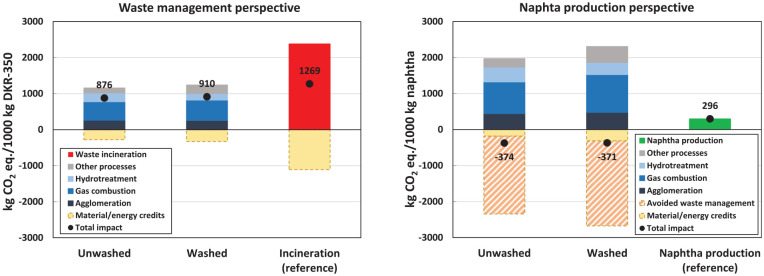
Cradle-to-gate lifecycle GHG emissions for waste management perspective (left) and naphtha production perspective (right). GHG: greenhouse gas.

In the waste management perspective, the results show substantial lifecycle GHG emission savings for pyrolysis compared to incineration with energy recovery, which serves as the reference system (Figure 3). The emission savings represent 31% and 28% for the unwashed and washed cases, respectively. Notably, the unwashed case indicates the highest reduction, resulting in estimated lifecycle GHG emissions of 876 kg CO_2_ eq. per tonne of DKR-350. When comparing the unwashed and washed cases, it becomes evident that the washing process does not yield substantial lifecycle GHG emission savings. Although the washed case demonstrates fewer emissions in the hydrotreatment step because of reduced hydrogen demand for the pyrolysis oil upgrade, these savings are offset by the additional energy consumption in the washing step.

Regarding the overall process contribution to the lifecycle GHG emissions of pyrolysis, the primary processes are pyrolysis gas combustion, agglomeration and hydrotreatment, accounting for 81%–88% of the gross impacts (i.e. impacts excluding credits). Remarkably, the combustion of pyrolysis gas contributes to 45% of the gross lifecycle GHG emissions (excluding credits). The GHG emissions from the agglomeration process are associated with electricity used to operate the agglomeration process line. In the case of hydrotreatment, most impacts originate from the hydrogen used in this process, as we assume the hydrogen is produced from natural gas.

Both the pyrolysis and reference systems include credits for avoiding specific products. Notably, credits for avoiding naphtha production substituted by upgraded pyrolysis oil account for 62% and 48% of the total credits in the unwashed and washed cases, respectively. The remaining credits are primarily given for the excess heat generated from pyrolysis gas combustion, which substitutes avoided Dutch market heat for industrial use (see the detailed dataset in the Supplemental Material). Credits for avoiding lignite by substituting the solid pyrolysis fraction are minor. Without considering credits, the emissions for unwashed and washed pyrolysis cases range from 1156 to 1239 kg CO_2_ eq., leading to 48%–51% savings compared to the non-credited reference system.

#### Naphtha production perspective

In the naphtha production perspective, the results demonstrate net-negative lifecycle GHG emissions in both pyrolysis cases, ranging from −374 to −371 kg CO_2_ eq. per tonne of produced upgraded pyrolysis oil with a naphtha-like quality (Figure 3). By contrast, producing 1 tonne of fossil naphtha in the refinery (reference system) emits 296 kg CO_2_ eq.

The net-negative GHG emissions in the pyrolysis cases primarily originate from credits for avoiding waste management of DKR-350. These credits account for 88%–92% of the total credits in the unwashed and washed cases. The avoided waste management credits are calculated based on the avoided CO_2_ emissions resulting from DKR-350 incineration in the incineration plant. However, the avoidance of waste management imposes an additional burden on the system as the electricity and heat that would have been generated through incineration are now lost. Consequently, the GHG emissions associated with producing additional market electricity and heat are deducted from the total credits. Furthermore, credits are granted for recovered energy (apart from avoided waste management, such as excess heat from gas combustion) and lignite substitution. Similarly, as in the waste management perspective, the unwashed and washed cases show relatively similar total lifecycle GHG emissions, indicating minor emission savings due to the washing process. The relative process contributions to the total impacts (excluding credits) align with those observed in the waste management perspective.

It is essential to exercise caution when interpreting the results from the naphtha production perspective. The findings of the pyrolysis cases do not necessarily imply that pyrolysis technology has net-negative impacts on climate change. The analysis assumes that the DKR-350 waste stream enters the system without any environmental burdens. Accounting for the lifecycle GHG emissions associated with the previous lifecycle of DKR-350 could increase the total lifecycle GHG emissions of the pyrolysis cases.

### Carbon recovery efficiency

**Figure 4.**
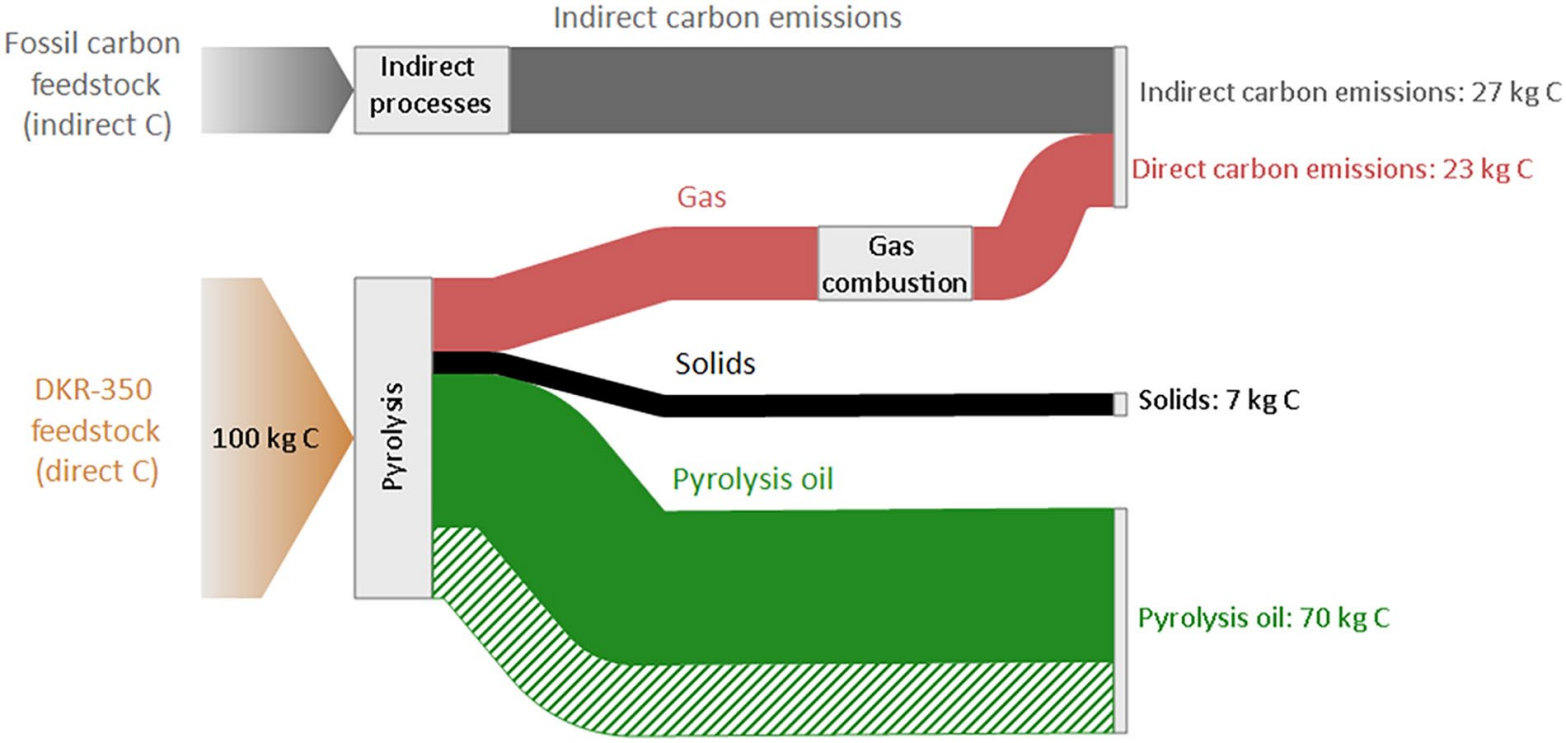
Carbon flow of the pyrolysis system, unwashed case. Data are normalised to 100 kg C in the feedstock to the pyrolysis reactor. The green dashed flow represents uncertain pyrolysis oil yield, assuming uncollected mass from the pilot-scale reactor is pyrolysis oil.

Figure 4 illustrates the carbon flows within the examined pyrolysis system in the unwashed case. The findings demonstrate that 48%–70% of the carbon input to pyrolysis is ultimately present in the pyrolysis oil, with the lower value in the range representing a conservative approach that only considers carbon in the collected pyrolysis oil. The remaining carbon from the pyrolysis feedstock is present in the form of gas or solids. Since this carbon is ultimately combusted (gas) or utilised as substitute energy products (solids), it is assumed that this carbon is emitted primarily as CO_2_. Furthermore, the results show that the pyrolysis system necessitates an additional 27 kg of carbon input in indirect processes to pyrolyse 100 kg of carbon in the DKR-350 feedstock. This indirect carbon flow accounts for carbon embedded in fossil fuels utilised during the indirect processes, predominantly for electricity generation, and represents approximately 21% of the total carbon input.

The overall carbon recovery efficiency of the system is estimated to be 38%–55%, denoting the proportion of total carbon recovered in the pyrolysis oil. It is crucial to note that this percentage does not signify the circularity level of the pyrolysis system, as the conversion of pyrolysis oil back to plastic polymer was not included in the assessment. The inclusion of this step would likely result in a lower overall carbon recovery efficiency. Furthermore, similar conclusions were found in the washed case, as washing had negligible impact on carbon flows in the pyrolysis system.

## Discussion

### Sensitivity analysis

Sensitivity analyses were conducted to quantify the effects of various uncertainties and alternative choices in order to better interpret the results. We identified three categories for our sensitivity analysis:

1. Model uncertainties: We examined how different modelling choices affect the results. Specifically, we analysed the sensitivity of two parameters: the application of the 50:50 approach and the assumption of lignite combustion (instead of its substitution).
2. Future perspective: We investigated the impact of the future electricity mix and improved waste management efficiencies for the year 2030; the changes in electricity mix and waste management efficiency affected the foreground processes.
3. Data uncertainties: We assessed the sensitivity of the data used in the LCA model, focusing on the two most uncertain data points: pyrolysis oil yield and pyrolysis reactor energy consumption. Additionally, we evaluated these data uncertainties within the 2030 future perspective.

Apart from the 50:50 approach, sensitivity analyses were performed in the context of the unwashed case within the waste management perspective. We expect that the conclusions derived from the sensitivity analyses that were not conducted for the washed case and the naphtha production perspective would align with those obtained from the performed sensitivity analysis.

#### Model uncertainties

##### The 50:50 approach

As defined by Corona et al. (2019), the 50:50 approach equally distributes the environmental burdens and credits from recycling between primary and recycled product cycles. In our case, this approach was not applicable because the production of primary products (i.e. cradle) was not included within our system boundaries. This exclusion was due to the impracticality of tracing the production of all materials in such a complex mixture as DKR-350. Instead, we adopted a modified 50:50 approach, where environmental burdens and credits were equally shared between the two primary products of the pyrolysis system: waste management and naphtha production. Under this modified approach, no credits are assigned for avoiding naphtha production in the waste management perspective and for avoiding DKR-350 waste management in the naphtha production perspective. System credits were only given (and shared) for by-products, such as excess heat and solid fractions.

Similarly, for waste incineration, environmental impacts are equally distributed between the two primary functions: waste management and energy production (heat and electricity). The 50:50 approach did not apply to the fossil naphtha production reference in the naphtha production perspective, as we used black box data with economic allocation where naphtha is the sole product of the system. In that case, we compared the pyrolysis’ 50:50 approach results in the naphtha production perspective to the baseline reference.

**Figure 5.**
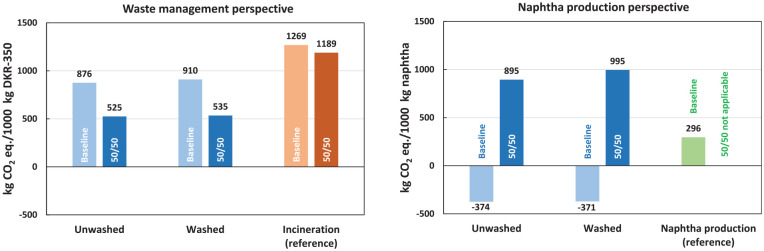
Sensitivity analysis for the 50:50 approach. Results are expressed in cradle-to-gate lifecycle GHG emissions from the waste management perspective (left) and the naphtha production perspective (right). GHG: greenhouse gas.

The results (Figure 5) demonstrate high sensitivity to applying the 50:50 approach within the LCA model. In the waste management perspective, adopting the 50:50 approach for pyrolysis led to a substantial 40%–41% reduction in lifecycle GHG emissions compared to the baseline. Consequently, the pyrolysis system achieved significantly greater GHG emission savings (55%–56%) compared to incineration (reference) than in the baseline scenario (28%–31%). A significant change was also observed from the naphtha production perspective, where lifecycle GHG emissions per 1000 kg of produced naphtha increased approximately threefold to fourfold within the pyrolysis system. With the 50:50 approach, lifecycle GHG emissions from naphtha production via pyrolysis surpassed those from fossil-based naphtha production.

In conclusion, the sensitivity analysis highlighted the importance of the 50:50 approach, as its application led to different outcomes and conclusions compared to the baseline for both perspectives. Thus, the selection of the modelling approach in LCA and the definition of system boundaries are critical parameters that significantly influence the results. While pyrolysis proves to be an advantageous waste management technology under the 50:50 approach, its effectiveness as a primary method for naphtha production appears less favourable from the standpoint of GHG emissions.

##### Lignite combustion

In the sensitivity analysis focused on lignite, we assessed the impact of our primary assumption that lignite is used outside our system boundaries, thereby earning credits for lignite substitution. This assumption was based on the fact that the heat produced from pyrolysis gas combustion is sufficient to sustain the pyrolysis process, with excess heat remaining. Therefore, there is no need for an additional energy source from solid combustion, allowing the solids to be sold and utilised in another facility or sector as an energy source.

Alternatively, we examined the impact on lifecycle GHG emissions if the solids were combusted within our system, with a thermal efficiency of 90%, and received credits for excess heat. The sensitivity analysis showed that if the solids were combusted under these conditions, the total lifecycle GHG emissions for both the unwashed and washed cases would increase by 11% compared to the baseline. Thus, the overall conclusions did not change, although the GHG savings of pyrolysis compared to the reference scenario would be smaller than in the baseline.

#### Future perspective

In this sensitivity analysis, the future perspective was focused on two key parameters which affected the foreground process within our system boundaries:

1. 2030 Electricity mix: We calculate the electricity mix in the Netherlands for 2030, employing the same methodology as in the baseline model. Data for the 2030 electricity supply are sourced from the Netherlands Climate and Energy Outlook 2021 report (PBL, 2021). Consequently, the new electricity mix was applied to the foreground system, which affected processes such as agglomeration or credits given for electricity.
2. Waste incinerator efficiency: We anticipate enhanced energy efficiency in future waste incinerators (CEWEP, 2022), with efficiency rates projected at 20.4% for electricity generation and 43.3% for heat generation. The changes in efficiencies affected the incineration process in the reference.

**Figure 6.**
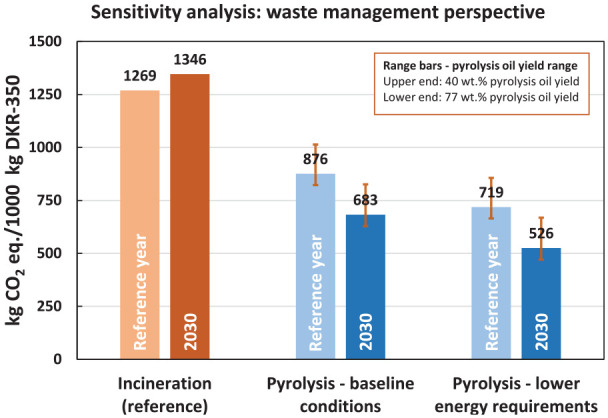
Results of the sensitivity analyses for the pyrolysis unwashed case, waste management perspective. Results are expressed in lifecycle cradle-to-gate GHG emissions (kg CO_2_ eq.) per 1000 kg DKR-350 waste management. GHG: greenhouse gas.

The sensitivity analysis results in Figure 6 show a 22% reduction in lifecycle GHG emissions, decreasing from 876 to 683 kg CO_2_ eq., as we alter the future perspective of the baseline model. This reduction is primarily attributable to the agglomeration process’s improved performance, facilitated by using less carbon-intensive electricity. On the contrary, the GHG emissions for the reference system increase from 1269 to 1346 kg CO_2_ eq. This shift occurs despite higher energy recovery efficiency in waste incineration, as fewer credits are given for the recovered, less carbon-intensive electricity. Consequently, substituting low-carbon renewable electricity with electricity derived from waste incineration does not yield higher GHG emission savings.

Comparing the lifecycle GHG emissions of the pyrolysis baseline to incineration in 2030, a substantial 49% reduction can be achieved. In contrast, the baseline model within the current temporal scope achieves only a 31% reduction. Thus, this sensitivity analysis underscores the critical influence of the electricity mix and its emission intensity on the overall lifecycle GHG emissions of the studied systems.

In the following sections, we investigated the impact of data uncertainties, specifically focusing on the effects of lower energy requirements for the pyrolysis process and variations in pyrolysis oil yield. This assessment was conducted for both the current temporal scope and the future 2030 perspective.

#### Data uncertainties

##### Lower energy requirements

In this sensitivity analysis, we examined the assumption concerning the energy requirements of the pyrolysis reactor. In our baseline model, we assumed that the pyrolysis energy requirement amounts to 15% of the caloric value of the DKR-350 feedstock. This assumption was grounded in empirical data from a technology provider, as elaborated in the ‘Pyrolysis’ section. However, as detailed above, the literature on mixed plastic waste pyrolysis also reports lower energy requirements, ranging from 3% to 8% of the feedstock’s calorific value. Consequently, the chosen pyrolysis energy requirement of 15% within the LCA model can be considered relatively conservative. Therefore, we conducted a sensitivity analysis considering a lower energy requirement of 6% of the feedstock’s calorific value. The value is based on process data from a pyrolysis plant processing 5000 tonnes of mixed plastic waste annually and drawn from an LCA study conducted by Jeswani et al. (2021).

Figure 6 illustrates that a 60% reduction in energy requirements, from 15% to 6% of the feedstock’s calorific input, leads to an additional 18% reduction in lifecycle GHG emissions (i.e. from 876 to 719 kg CO_2_ eq.). This yields a total lifecycle GHG emissions savings of 43% compared to incineration. Furthermore, combining reduced energy requirements with the 2030 future perspective results in substantial lifecycle GHG emission savings in the pyrolysis system, reducing them by 61% compared to incineration.

Notably, it is crucial to acknowledge that the extent of savings achieved through lower energy requirements depends upon credits derived from the surplus heat preserved due to reduced energy demand. Consequently, the pyrolysis reactor’s energy requirement does not emerge as a highly sensitive parameter. However, it is worth noting that further savings can be realised when energy requirements are minimised.

##### Pyrolysis oil yield

The mass balance of the studied pilot-scale pyrolysis was not fully closed, caused by incomplete mass collection at the pyrolysis reactor output. To close the mass balance in our LCA model, it was assumed that the produced pyrolysis oil supplemented the uncollected mass. Despite the uncertainty of this assumption, the overall pyrolysis oil yield did not surpass the yield observed in the lab-scale trials using a fixed-bed reactor (Genuino et al., 2022).

Furthermore, a sensitivity analysis was conducted to assess the uncertainty of the pyrolysis oil yield and its impact on lifecycle GHG emissions for the unwashed case in the waste management perspective. Lifecycle GHG emissions were re-calculated for optimistic and pessimistic cases:

1. Maximum oil yield: This case considered the highest pyrolysis oil yield achieved during the lab-scale trials (Genuino et al., 2022). The estimated yields for pyrolysis oil, gas and solids were 77 wt.%, 15 wt.% and 8 wt.%, respectively. The composition and calorific values of the products were assumed to be consistent with those measured in the pilot-scale trials. Given the inadequacy of the energy recovered from gas combustion to fulfil the energy demands of pyrolysis and hydrotreatment, an external heat supply was included in the system.
2. Conservative oil yield: In this case, the remaining uncollected mass was supplemented with additional pyrolysis gas yield. We assumed that the additional theoretical gas possessed the same composition and calorific value as the collected gas. The product yields considered in the analysis were 40 wt.% for pyrolysis oil, 53 wt.% for gas and 7 wt.% for solids.

We expect that the reality could possibly lie between these two cases. The sensitivity analysis for the pyrolysis oil yield shown in Figure 6 (see range bars) demonstrates that the pyrolysis (in all studied cases) yielded lower GHG emissions than DKR-350 incineration in both the maximum and conservative sensitivity analysis cases.

For the pyrolysis baseline case, the reduction in lifecycle GHG emissions compared to the reference ranges between 20% (conservative oil yield) and 35% (maximum oil yield). Despite the highly conservative assumption regarding the oil yield in the conservative case (only 40 wt.% pyrolysis oil yield), pyrolysis still outperformed incineration with energy recovery. However, when comparing the maximum and conservative oil yield cases, increasing the pyrolysis oil yield nearly twofold (from 40 wt.% in the conservative yield case to 77 wt.% in the maximum yield case) only resulted in an additional 15% reduction in GHG emissions relative to incineration. This relatively modest reduction is due to trade-offs within the lifecycle processes. For example, in the maximum oil yield case, an external heat supply was needed for pyrolysis because the low gas yield was insufficient to meet the energy requirements of the pyrolysis reactor. Additionally, achieving a higher oil yield required more process energy to upgrade the pyrolysis oil during the hydrotreatment step. Consequently, the increase in process energy and the associated GHG emissions offset the savings gained from the higher oil yield.

The pyrolysis oil yield parameter was also studied under the 2030 future perspective, considering lower energy requirements and their combination (see Figure 6). The highest savings compared to incineration were observed in the combined case of lower energy requirements for pyrolysis and the future perspective. In this case, lifecycle GHG savings ranged between 50% (conservative oil yield) and 65% (maximum oil yield). This pronounced reduction relative to incineration underscores the potential of pyrolysis as a future waste management technology while highlighting opportunities for further technological development and improvement.

In conclusion, the sensitivity analysis on pyrolysis oil yield indicates that, in this particular case, the oil yield is not a highly sensitive parameter regarding GHG emission savings when considered alone due to trade-offs within the lifecycle processes. Nevertheless, the results show that the highest oil yield, combined with other improved process parameters, delivers the most favourable performance among the examined cases. Additionally, from the perspective of circular economy objectives, a higher oil yield is desirable as it enhances the system’s carbon recovery efficiency.

### Towards carbon circularity

Our analysis of carbon recovery efficiency indicated potential trade-offs between the environmental and circularity performance of pyrolysis. This analysis included an essential aspect: assessing these trade-offs by accounting for indirect carbon flows caused by background processes, primarily attributed to carbon emissions from energy production. When these flows are accounted for, the overall carbon recovery efficiency ranges from 38% to 55%, with the former figure referring to a lower pyrolysis oil yield and the latter figure referring to the baseline model. Further analysis revealed that the carbon recovery efficiency of the pyrolysis system could increase to 40%–59% within the future perspective in 2030. This improvement is attributed to the use of less carbon-intensive electricity, which consequently reduces overall indirect carbon emissions. This underscores the critical role of energy and its associated carbon intensity in achieving enhanced carbon recovery efficiency. In summary, to increase carbon circularity in the plastics sector, indirect carbon flows must be reduced.

Nevertheless, pilot-scale trials and our analysis indicated that even with a decrease in indirect carbon flow, it is unfeasible for pyrolysis to achieve complete carbon circularity for plastics due to the thermochemical equilibrium reached in the reactor unless the gaseous and solid products are utilised for purposes other than energy recovery. Additionally, it is essential to acknowledge that our analysis did not account for potential additional carbon losses that may arise if system boundaries are expanded to include polymer production derived from pyrolysis oil, potentially resulting in even lower carbon recovery efficiency.

Despite pyrolysis being a promising circular technology capable of handling mixed plastic waste that would otherwise be incinerated, its role as a standalone circular strategy is constrained within the context of carbon circularity for the aforementioned reasons. Consequently, to close the carbon loop, carbon recovery through pyrolysis must be complemented by other carbon sources within the circular economy, such as carbon from sustainably cultivated biomass. Lastly, as Stegmann et al. (2022) concluded in their analysis that realising a fully circular plastics sector remains challenging for any circular strategy as long as the current growing demand for plastics persists.

### Comparison with other studies

The LCA outcomes and the accompanying sensitivity analyses conducted in this study reveal that the pyrolysis system displays high sensitivity to various variables, including system boundaries, feedstock composition, pyrolysis reactor performance, geographical and temporal scope (e.g. influenced by the carbon-intensity of the electricity mix). Consequently, comparing the results with other LCA studies on pyrolysis proves challenging. Furthermore, our ability to make comparisons is constrained to the waste management perspective, as no equivalent studies assessed naphtha as a primary product. For instance, Jeswani et al. (2021) and Somoza-Tornos et al. (2020) carried out LCAs from product perspectives; however, they determined low-density polyethylene (LDPE) granulate and ethylene as the main products for their systems, respectively.

A study by Jeswani et al. (2021) provides the closest correspondence to the scope of our investigation in the waste management perspective. The authors calculated lifecycle GHG emissions for a pyrolysis system with similar pyrolysis oil yield (71 wt.% compared to our 68 wt.% in the baseline model) at 739 kg CO_2_ eq., showing a better pyrolysis performance than in our model. However, their baseline analysis assumed Germany’s electricity mix in 2030. Considering pyrolysis lifecycle GHG emissions from the baseline case in 2030, our results are in a similar range as that reported by them.

In a recent study by Civancik-Uslu et al. (2021), mixed polyolefin waste was thermochemically recycled in a case study focused on Belgium, yielding GHG emissions of 539 kg CO_2_ eq. per metric tonne of waste. Although this value is lower than our baseline case, it remains within the range of our results, particularly in the context of the most optimal scenario.

Lastly, in the same geographical scope as our study and utilising the same feedstock, a screening LCA conducted by CE Delft (2019) produced significantly different outcomes, revealing net-negative lifecycle GHG emissions per metric tonne of waste-managed DKR-350. Unfortunately, this study lacks a detailed breakdown of the lifecycle process, making it impossible to identify the critical disparities compared to our results.

### Limitations

The conducted environmental impact assessment of DKR-350 pyrolysis with a focus on lifecycle GHG emissions provided a detailed analysis of the studied pyrolysis systems. However, the study has certain limitations and uncertain assumptions, as discussed below.

One of the primary limitations stems from utilising a pilot-scale pyrolysis reactor primarily designed for ex-situ catalytic biomass pyrolysis rather than for non-catalytic pyrolysis of plastics. As such, aside from the pyrolysis reactor, the design also entailed a second reactor (typically for catalytic upgrading), which cannot be by-passed in the present work with mixed plastic waste. Thus, the pyrolysis system operates with a higher residence time (about 2 minutes, e.g. as compared to the previous lab-scale experiments), which may directly influence the distribution of products (between oil and gas) and the formation of aromatic products. Similar observations regarding the reactor design parameters were also reported by Kusenberg et al. (2022). Furthermore, the mass balance in the pyrolysis process was closed at 72–80 wt.%, with the remaining mass uncollected and unanalysed. In this case, an assumption was made to estimate the characteristics of the uncollected products by assuming that it consisted of oil with the same properties as the analysed collected oil. Nevertheless, the results suggest a potential research gap to investigate the influence of pyrolysis reactor design on reaction products and optimise the pyrolysis process to achieve the highest mass yield for pyrolysis oil.

Another study limitation arises from the LCA methodology, which has limitations when applied to recycling systems. The approach used in this study does not consider the previous life cycle of DKR-350, assuming that the waste stream enters the system without any environmental burdens. This assumption directly affects the results, as observed in the net-negative lifecycle GHG emissions of pyrolysis results in the naphtha production perspective. A similar observation can be seen in the LCA study by Jeswani et al. (2021). The study revealed net-negative lifecycle GHG emissions within a product-oriented perspective, similar to our naphtha production perspective. To gain deeper insights into these net-negative lifecycle GHG emissions associated with pyrolysis and other recycling technologies when adopting a product-oriented LCA goal, future research could prioritise evaluating pyrolysis systems through a consequential LCA approach. This approach necessitates an extension of the system boundaries to account for the environmental consequences of the innovation.

Another limitation of the applied LCA methodology arises from substituting pyrolysis oil with naphtha. A previous study indicated that only 27 wt.% of pyrolysis oil derived from DKR-350 falls within the boiling range of naphtha (i.e. <200°C), while the remainder aligns more closely with the boiling points of transportation fuels such as kerosene and diesel (Genuino et al., 2022). Consequently, an additional cracking step would be necessary to achieve the boiling range of naphtha, leading to an increased impact on the pyrolysis system due to the lifecycle GHG emissions from the cracking process. However, the cracking of pyrolysis oil was not included within our system boundaries, and its impact was not quantified due to the limited data availability for this process.

Similarly, a limitation in our study regarding the process design is the potential underestimation of the complexity involved in further upgrading pyrolysis oil during the hydrotreatment step, as the data were unavailable. This is particularly relevant to the impacts of impurities on product quality, yield and processing costs.

Lastly, our analysis evaluated lifecycle GHG emissions as the only LCA indicator. While GHG emissions serve as a crucial and frequently paramount metric for assessing the efficiency of circular economy strategies in mitigating climate change impacts, future LCA studies on pyrolysis and plastics circularity could be enriched by incorporating other LCA indicators, notably toxicity. Including such additional indicators would contribute to a more comprehensive understanding of the environmental sustainability of pyrolysis, offering a broader perspective on its ecological impact beyond the scope of GHG emissions.

## Conclusions

In this study, we explored the lifecycle GHG emissions associated with the pyrolysis of DKR-350. Our findings revealed substantial potential for reducing lifecycle GHG emissions through pyrolysis of unwashed DKR-350, with savings ranging from 20% to 48% compared to incineration with energy recovery within the current temporal scope. In the context of a 2030 outlook, these savings could range from 39% to 65%. Additionally, our analysis showed that the pre-treatment of DKR-350 via washing does not yield significant lifecycle GHG emission savings compared to the unwashed case. The analysis was carried out based on empirical data, which is currently underreported in the literature on LCA case studies.

Furthermore, we assessed different methodological choices to access circular carbon utilisation within the LCA framework. We analysed the case study from two perspectives of the pyrolysis system: waste management and naphtha production. Our analysis showed high sensitivity of the results to these methodological choices. By presenting different perspectives on waste management, we highlight the importance of incorporating diverse approaches when addressing the role of pyrolysis as a waste management tool for circularity. Moreover, these insights are key to understanding the circularity performance measurements using the LCA framework, particularly for plastic recycling.

Next to assessing the environmental sustainability indicator of lifecycle GHG emissions, we included the carbon recovery efficiency indicator in our study to quantify the system’s carbon circularity. We calculated the carbon recovery efficiency of the pyrolysis system, which falls within the range of 38%–55% of the total recovered carbon in the pyrolysis oil, with the former figure referring to a lower pyrolysis oil yield and the latter figure referring to the baseline model. Although carbon recovery efficiency does not represent the final level of carbon circularity for plastics in our case study – since full plastics-to-plastics conversion was beyond the scope of our research – it remains a valuable indicator of the technology’s potential to recover carbon for further utilisation. Based on our findings, we strongly advocate for incorporating the carbon recovery efficiency calculation approach employed in our study, which includes indirect carbon emissions, into any comprehensive circularity and efficiency assessment on plastics circularity. Moreover, we emphasise the need for an integrated approach to circular strategies that consider their broader sustainability implications, advocating for improved indicators to measure circularity and sustainability beyond conventional metrics. This recommendation aims to enhance the accuracy and comprehensiveness of policy considerations in plastics circularity assessments and provide a more nuanced and realistic reflection on the impact of circularity strategies.

In conclusion, this study finds that DKR-350 pyrolysis demonstrates promise as a relatively carbon-efficient waste management solution, with opportunities for further improvements in sustainability. The role of pyrolysis in advancing carbon circularity emerges as a complex yet critical technology in achieving circularity goals. Despite the challenges in the technological development of pyrolysis that have not been overcome yet, it offers promising potential as a future-oriented technology for managing hard-to-recycle waste, mitigating GHG emissions, preserving carbon in the carbon loop and thus reducing dependence on the fossil feedstock for the plastic sector.

## Supplemental Material

sj-docx-1-wmr-10.1177_0734242X241306605 – Supplemental material for Pyrolysis of Dutch mixed plastic waste: Lifecycle GHG emissions and carbon recovery efficiency assessment Supplemental material, sj-docx-1-wmr-10.1177_0734242X241306605 for Pyrolysis of Dutch mixed plastic waste: Lifecycle GHG emissions and carbon recovery efficiency assessment by Juraj Petrík, Homer C. Genuino, Gert Jan Kramer and Li Shen in Waste Management & Research
