# Supplementary material for: Pyrolysis of Dutch mixed plastic waste: Lifecycle GHG emissions and carbon recovery efficiency assessment
Source: Waste Manag Res. 2024 Dec 31;43(8):1219–33. doi: 10.1177/0734242X241306605 (PMC12301508; doi:10.1177/0734242X241306605)
Supplement: sj-docx-1-wmr-10.1177_0734242X241306605 – Supplemental material for Pyrolysis of Dutch mixed plastic waste: Lifecycle GHG emissions and carbon recovery efficiency assessment [file sj-docx-1-wmr-10.1177_0734242X241306605.docx]

**Supplementary Information**

**A. Life cycle inventory**

*Table S.1. LCI for DKR-350 pyrolysis, unwashed case.*

|  | **Waste management perspective** | **Naphtha production perspective** | **Unit** | **ecoinvent dataset (or other environmental profile source)** |
| --- | --- | --- | --- | --- |
| **Shredding** |  |  |  |  |
| *Input* |  |  |  |  |
| DKR-350, wet mass | 1000 | 1705 | kg |  |
| Electricity | 77 | 132 | kWh | See Table S.3 |
|  |  |  |  |  |
| *Output* |  |  |  |  |
| DKR-350 flakes, wet mass | 1000 | 1705 | kg |  |
| **Agglomeration** |  |  |  |  |
| *Input* |  |  |  |  |
| DKR-350 flakes, wet mass | 1000 | 1705 | kg |  |
| Electricity | 648 | 1104 | kWh | See Table S.3 |
|  |  |  |  |  |
| *Output* |  |  |  |  |
| DKR-350 agglomerate, dry mass | 849 | 1447 | kg |  |
| Moisture removal | 140 | 239 | kg |  |
| Metals removal | 11 | 19 | kg |  |
| **Pyrolysis** |  |  |  |  |
| *Input* |  |  |  |  |
| DKR-350 agglomerate, dry mass | 849 | 1447 | kg |  |
| Energy requirements | 5224 | 8905 | MJ | supplied from combusted gas |
| Nitrogen (g) | 477 | 814 | kg | Nitrogen, liquid {RER}\| air separation, cryogenic \| Cut-off, U |
|  |  |  |  |  |
| *Output* |  |  |  |  |
| Crude p. oil | 577 | 983 | kg |  |
| Solids | 56 | 96 | kg |  |
| Gas | 216 | 368 | kg |  |
|  |  |  |  |  |
| **Gas combustion** |  |  |  |  |
| *Input* |  |  |  |  |
| Gas | 216 | 368 | kg |  |
|  |  |  |  |  |
| *Output* |  |  |  |  |
| Recovered heat | 7287 | 12442 | MJ |  |
| CO_2_ emissions | 516 | 880 | kg |  |
| **Hydrotreatment** |  |  |  |  |
| *Input* |  |  |  |  |
| Crude p. oil | 577 | 983 | kg |  |
| Hydrogen | 20 | 34 | kg | (Fernández-Dacosta et al., 2019) |
| Electricity | 53 | 91 | kWh | See Table S.3 |
|  |  |  |  |  |
| *Output* |  |  |  |  |
| Naphtha-quality pyrolysis oil | 587 | 1000 | kg |  |
| Removed contaminants | 10 | 18 | kg |  |
| **Substitution** |  |  |  |  |
| Avoided naphtha | 587 | - | kg | Naphtha {RER}\| market for \| Cut-off, U |
| Avoided lignite | 151 | 258 | kg | Lignite {RER}\| market for \| Cut-off, U |
| Avoided heat (gas combustion) | 2063 | 3517 | MJ | Heat, district or industrial, natural gas {Europe without Switzerland}\| market for heat, district or industrial, natural gas \| Cut-off, U |
| Avoided waste management | - | 1705 | kg |  |

*Table S.2. LCI for DKR-350 pyrolysis, washed case.*

|  | **Waste management perspective** | **Naphtha production perspective** | **Unit** | **Ecoinvent dataset (or other environmental profile source)** |
| --- | --- | --- | --- | --- |
| **Shredding** |  |  |  |  |
| *Input* |  |  |  |  |
| DKR-350, wet mass | 1000 | 1861 | kg |  |
| Electricity | 77 | 144 | kWh | See Table S.3 |
|  |  |  |  |  |
| *Output* |  |  |  |  |
| DKR-350 flakes, wet mass | 1000 | 1861 | kg |  |
| **Washing** |  |  |  |  |
| *Input* |  |  |  |  |
| DKR-350 flakes, wet mass | 1000 | 1861 | kg |  |
| Water | 2500 | 4652 | kg | Tap water {RER}\| market group for \| Cut-off, U |
| Detergent | 14 | 26 | kg | Soap {RER}\| production \| Cut-off, U |
| NaOH | 25 | 47 | kg | Sodium hydroxide, without water, in 50% solution state {GLO}\| market for \| Cut-off, U |
| Electricity | 100 | 186 | kWh | See Table S.3 |
|  |  |  |  |  |
| *Output* |  |  |  |  |
| Washed DKR-350 flakes, wet mass | 990 | 1841 | kg |  |
| Wastewater | 2550 | 4744 | kg | Wastewater, average {Europe without Switzerland}\| market for wastewater, average \| Cut-off, U |
| **Agglomeration** |  |  |  |  |
| *Input* |  |  |  |  |
| Washed DKR-350 flakes, wet mass | 990 | 1841 | kg |  |
| Electricity | 641 | 1193 | kWh | See Table S.3 |
|  |  |  |  |  |
| *Output* |  |  |  |  |
| DKR-350 agglomerate, dry mass | 835 | 1553 | kg |  |
| Moisture removal | 140 | 261 | kg |  |
| Metals removal | 15 | 28 | kg |  |
|  |  |  |  |  |
| **Pyrolysis** |  |  |  |  |
| *Input* |  |  |  |  |
| DKR-350 agglomerate, dry mass | 835 | 1553 | kg |  |
| Energy requirements | 4907 | 9131 | MJ | supplied from combusted gas |
| Nitrogen (g) | 469 | 874 | kg | Nitrogen, liquid {RER}\| air separation, cryogenic \| Cut-off, U |
|  |  |  |  |  |
| *Output* |  |  |  |  |
| Crude p. oil | 536 | 998 | kg |  |
| Solids | 62 | 116 | kg |  |
| Gas | 236 | 439 | kg |  |
| **Gas combustion** |  |  |  |  |
| *Input* |  |  |  |  |
| Gas | 236 | 439 | kg |  |
|  |  |  |  |  |
| *Output* |  |  |  |  |
| Recovered heat | 8235 | 15324 | MJ |  |
| CO_2_ emissions | 563 | 1048 | kg |  |
| **Hydrotreatment** |  |  |  |  |
| *Input* |  |  |  |  |
| Crude p. oil | 536 | 998 | kg |  |
| Hydrogen | 15 | 27 | kg | (Fernández-Dacosta et al., 2019) |
| Electricity | 49 | 92 | kWh | See Table S.3 |
|  |  |  |  |  |
| *Output* |  |  |  |  |
| Naphtha-quality pyrolysis oil | 537 | 1000 | kg |  |
| Removed contaminants | 14 | 25 | kg |  |
| **Substitution** |  |  |  |  |
| Avoided naphtha | 537 | - | kg | Naphtha {RER}\| market for \| Cut-off, U |
| Avoided lignite | 152 | 282 | kg | Lignite {RER}\| market for \| Cut-off, U |
| Avoided heat (gas combustion) | 3328 | 6193 | MJ | Heat, district or industrial, natural gas {Europe without Switzerland}\| market for heat, district or industrial, natural gas \| Cut-off, U |
| Avoided waste management | - | 1861 | kg |  |

*Table S.3. LCI for Dutch electricity mix 2020 & 2030, production of 1kWh high voltage electricity (Electricity, high voltage {NL}| market for | Cut-off, U). The dataset is further implemented within the Electricity, medium voltage {NL}| market for | Cut-off, U dataset. Values are expressed in kWh per kWh produced electricity. Source: (PBL, 2021).*

| **Updated ecoinvent dataset**  *Electricity, high voltage {NL}\| market for \| Cut-off, U* | **Year 2020** | **Year 2030** |
| --- | --- | --- |
| Electricity, high voltage {NL}\| electricity production, natural gas, combined cycle power plant \| Cut-off, U | 0,251 | 0,111 |
| Electricity, high voltage {NL}\| electricity production, natural gas, conventional power plant \| Cut-off, U | 0,009 | 0,004 |
| Electricity, high voltage {NL}\| heat and power co-generation, natural gas, combined cycle power plant, 400MW electrical \| Cut-off, U | 0,172 | 0,076 |
| Electricity, high voltage {NL}\| heat and power co-generation, natural gas, conventional power plant, 100MW electrical \| Cut-off, U | 0,157 | 0,070 |
| Electricity, high voltage {NL}\| electricity production, hard coal \| Cut-off, U | 0,024 | 0,000 |
| Electricity, high voltage {NL}\| heat and power co-generation, hard coal \| Cut-off, U | 0,037 | 0,000 |
| Electricity, high voltage {NL}\| treatment of blast furnace gas, in power plant \| Cut-off, U | 0,032 | 0,004 |
| Electricity, high voltage {NL}\| electricity production, nuclear, pressure water reactor \| Cut-off, U | 0,034 | 0,023 |
| Electricity, high voltage {NL}\| electricity production, wind, 1-3MW turbine, onshore \| Cut-off, U | 0,080 | 0,080 |
| Electricity, high voltage {NL}\| electricity production, wind, 1-3MW turbine, offshore \| Cut-off, U | 0,045 | 0,432 |
| Electricity, low voltage {NL}\| electricity production, photovoltaic, 570kWp open ground installation, multi-Si \| Cut-off, U | 0,066 | 0,162 |
| Electricity, high voltage {NL}\| heat and power co-generation, wood chips, 6667 kW, state-of-the-art 2014 \| Cut-off, U | 0,064 | 0,019 |
| Electricity, high voltage {NL}\| heat and power co-generation, biogas, gas engine \| Cut-off, U | 0,008 | 0,002 |
| Electricity, high voltage {NL}\| market for \| Cut-off, U *(“Overig” in the PBL report, calculated as average of above technologies)* | 0,020 | 0,016 |
| Electricity, high voltage {NL}\| market for \| Cut-off, U *(transmission losses)* | 0,018 | 0,018 |

**B. DKR-350 characterization**

The raw data on DKR-350 composition were provided by NTCP. Further, data were adjusted according to the following assumptions:

- Polymers from the same material group are aggregated under one material (e.g. PE-film (non-black and black) and PE-rigid (non-black and black) are aggregated under PE).
- Composition of the “clogged” fraction (provided by NTCP):
  - LDPE: 41,99 wt.%
  - HDPE: 0 wt.%
  - PP: 20,61 wt.%
  - PS: 1,44 wt.%
  - Multi-layers: 21,39 wt.%
  - PET: 3,54 wt.%
  - Metals: 2,23 wt.%
  - Paper and cardboard: 8,79 wt.%
- Multi-layers are assumed to be composed of: 70 wt.% PE, 30 wt.% paper.
- Composition of “compound” assumed to be the same as “multi-layers”.
- “Fine fraction” is assumed to be composed of 100 wt.% PE.
- Moisture content of 14 wt.% is assumed for all DKR-350 batches.

*Table S.4. Characterization of DKR-350 batches used in pyrolysis. Source (HHV and C content): Phyllis2 database.*

| **Material** | **Composition [wt.%]** | | **HHV**  **[MJ/kg]** | **C content**  **[wt.%]** |
| --- | --- | --- | --- | --- |
|  | **Batch 1**  **(unwashed case)** | **Batch 2**  **(washed case)** |  |  |
| PE | 45,43 | 41,50 | 47,30 | 85,60 |
| PP | 17,80 | 17,33 | 47,30 | 85,60 |
| PS | 3,33 | 1,33 | 42,40 | 92,30 |
| PET | 6,99 | 11,03 | 22,93 | 62,64 |
| PVC | 0,01 | 0,00 | 17,80 | 38,40 |
| Metals | 1,14 | 1,49 | - | - |
| Paper and cardboard | 8,42 | 7,92 | 19,37 | 47,30 |
| Textile | 0,32 | 1,37 | 18,87 | 44,40 |
| Other | 1,51 | 2,97 | - | - |
| Organics | 1,05 | 1,05 | 19,71 | 52,09 |
| Moisture | 14,00 | 14,00 | - | - |
| Total | 100 | 100 |  |  |
| *HHV DKR-350 [MJ/kg]* | *34,82* | *32,92* |  |  |
| *Carbon content DKR-350 [kg C/kg]* | *0,66* | *0,63* |  |  |

**C. Pyrolysis gas characterisation**

*Table S.5. Characterisation of pyrolysis gas. The table shows calculated values for HHV and carbon content; for calculations, ideal gas behaviour was assumed at the temperature of 273,15 K and pressure of 101325 Pa. Source (HHV): (Mištová et al., 2016).*

| **Pyrolysis gas composition** | **Composition [wt.%]** | | **HHV**  **[MJ/kg]** | **C content**  **[wt.%]** |
| --- | --- | --- | --- | --- |
|  | **Unwashed** | **Washed** |  |  |
| H_2_ | 2,23 | 3,13 | 142,44 | 0,00 |
| CO | 6,64 | 8,06 | 10,22 | 0,43 |
| CO_2_ | 24,52 | 22,49 | 0,00 | 0,27 |
| CH_4_ | 9,99 | 10,22 | 55,76 | 0,75 |
| ethylene | 15,62 | 16,99 | 50,76 | 0,86 |
| ethane | 3,58 | 3,52 | 51,69 | 0,80 |
| propylene | 30,86 | 28,56 | 49,28 | 0,86 |
| propane | 4,16 | 3,74 | 50,41 | 0,82 |
| benzene | 2,11 | 3,04 | 41,99 | 0,92 |
| toluene | 0,29 | 0,25 | 42,55 | 0,91 |
|  |  |  |  |  |
| HHV gas [MJ/kg] | 37,52 | 38,77 |  |  |
| C content in gas [kg C/kg] | 0,65 | 0,65 |  |  |

**Bibliography**

Fernández-Dacosta, C., Shen, L., Schakel, W., Ramirez, A., & Kramer, G. J. (2019). Potential and challenges of low-carbon energy options: Comparative assessment of alternative fuels for the transport sector. *Applied Energy*, *236*(November 2018), 590–606. https://doi.org/10.1016/j.apenergy.2018.11.055

Mištová, E., Macák, J., & Jelínek, L. (2016). *Energetika - Návody k výpočtům* (2nd ed.). VŠCHT.

PBL. (2021). *Klimaat- en Energieverkenning 2021*.
